# The Increased Expression of Regulator of G-Protein Signaling 2 (RGS2) Inhibits Insulin-Induced Akt Phosphorylation and Is Associated with Uncontrolled Glycemia in Patients with Type 2 Diabetes

**DOI:** 10.3390/metabo11020091

**Published:** 2021-02-05

**Authors:** J. Gustavo Vazquez-Jimenez, M. Stephanie Corpus-Navarro, J. Miguel Rodriguez-Chavez, Hiram J. Jaramillo-Ramirez, Judith Hernandez-Aranda, Octavio Galindo-Hernandez, J. Rene Machado-Contreras, Marina Trejo-Trejo, Agustin Guerrero-Hernandez, J. Alberto Olivares-Reyes

**Affiliations:** 1 Department of Biochemistry, Center for Research and Advanced Studies of the National Polytechnic Institute, CINVESTAV-IPN, Mexico City 07360, Mexico; gustavo.vazquez@uabc.edu.mx (J.G.V.-J.); juhernandez@cinvestav.mx (J.H.-A.); aguerrero@cinvestav.mx (A.G.-H.); 2 Laboratory of Molecular Pathogenesis, School of Medicine, Campus Mexicali, Autonomous University of Baja California, Mexicali, Baja California 21000, Mexico; stephanie.corpus@uabc.edu.mx (M.S.C.-N.); jesusm.rodriguez@udem.edu (J.M.R.-C.); rene.machado@uabc.edu.mx (J.R.M.-C.); 3 Department of Internal Medicine, General Hospital of Mexicali, Mexicali, Baja California 21000, Mexico; jaramilloh@uabc.edu.mx; 4 Laboratory of Biochemistry, School of Medicine, Campus Mexicali, Autonomous University of Baja California, Mexicali, Baja California 21000, Mexico; octavio.galindo@uabc.edu.mx; 5 School of Sports, Campus Mexicali, Autonomous University of Baja California, Mexicali, Baja California 21000, Mexico; marina.trejo@uabc.edu.mx

**Keywords:** Akt protein, HbA1c, human endothelial cells, insulin resistance, palmitic acid, regulator of G-protein signaling 2, type 2 diabetes mellitus

## Abstract

Experimental evidence in mice models has demonstrated that a high regulator of G-protein signaling 2 (RSG2) protein levels precede an insulin resistance state. In the same context, a diet rich in saturated fatty acids induces an increase in RGS2 protein expression, which has been associated with decreased basal metabolism in mice; however, the above has not yet been analyzed in humans. For this reason, in the present study, we examined the association between RGS2 expression and insulin resistance state. The incubation with palmitic acid (PA), which inhibits insulin-mediated Akt Ser^473^ phosphorylation, resulted in the increased RGS2 expression in human umbilical vein endothelial-CS (HUVEC-CS) cells. The RGS2 overexpression without PA was enough to inhibit insulin-mediated Akt Ser^473^ phosphorylation in HUVEC-CS cells. Remarkably, the platelet RGS2 expression levels were higher in type 2 diabetes mellitus (T2DM) patients than in healthy donors. Moreover, an unbiased principal component analysis (PCA) revealed that RGS2 expression level positively correlated with glycated hemoglobin (HbA1c) and negatively with age and high-density lipoprotein cholesterol (HDL) in T2DM patients. Furthermore, PCA showed that healthy subjects segregated from T2DM patients by having lower levels of HbA1c and RGS2. These results demonstrate that RGS2 overexpression leads to decreased insulin signaling in a human endothelial cell line and is associated with poorly controlled diabetes.

## 1. Introduction

Insulin resistance is a systemic disorder in which cells fail to respond to normal levels of circulating insulin. Under this condition, insulin’s highly critical metabolic functions, mainly on hepatic, muscular, and adipose tissues, such as glucose uptake and synthesis of glycogen, lipids, and proteins, are altered [1]. Experimental and clinical trials have provided evidence that insulin resistance in metabolic tissues constitutes a characteristic feature of metabolic dysfunction, mainly induced by obesity [1]. This peripheral insulin resistance causes pancreatic β-cells to secrete more insulin, in a process known as compensatory hyperinsulinemia. However, together with insulin resistance, there is often a β-cell reduction, resulting in sustained hyperglycemia and type 2 diabetes mellitus (T2DM) [2,3]. At the molecular level, insulin resistance is the consequence of insulin signaling impairment resulting from mutations or post-translation modification of the insulin receptor (IR) itself or any of its downstream effectors, including the insulin receptor substrate (IRS) proteins, PI3K, and Akt [1,4]. Particularly, the reduction of insulin-stimulated Akt phosphorylation of serine 473 (Ser^473^) is considered a reliable indicator of insulin resistance [4,5]. Moreover, we have recently shown that palmitic acid (PA) but not palmitoleic acid (POA) induces insulin resistance by decreasing insulin-induced Akt phosphorylation in human umbilical endothelial cells [6].

Regulator of G-protein signaling (RGS) proteins terminate G-protein signaling by accelerating GTPase activity of heterotrimeric Gα-subunits [7]. RGS2 is one of the most widely studied proteins of the RGS family and preferentially inhibits signaling mediated by heterotrimeric G_q/11_ proteins [8,9] and has been associated with critical physiological processes such as regulation of blood pressure, cardiac remodeling, and immune responses [10]. As a critical regulator of G protein-coupled receptor (GPCR) signaling, its deficiency or low expression under experimental or pathological conditions severely affects important physiological conditions. RGS2 deficiency, for example, accelerates the progression of kidney fibrosis [11], enhances β-cell apoptosis and dysregulate insulin secretion in response to a glucose challenge [12], and promotes severe adipogenesis and lipolysis in brown adipose tissue [9]. Additionally, RGS2 upregulation attenuates phenylephrine hypertrophic effects via α_1_-adrenergic receptors in cultured ventricular myocytes [13].

RGS2 is also involved in regulating insulin activity in animal models. An RGS2 knockout mouse (*rgs2^−/−^*) was resistant to gain weight with age and showed increased insulin sensitivity [14]. Nevertheless, this higher insulin signaling in KO mice do not prevail in the presence of a high caloric intake since feeding a high-fat diet (HFD) for 25 weeks develops similar insulin resistance in both *rgs2^−/−^* and wild-type mice. HFD increases the expression of RGS2 in liver and white adipose tissue, but it decreases its heart expression [14]. This report suggests that increased RGS2 protein level associates with an insulin resistance state in mice. On the other hand, a study in a mice insulinoma cell line (βTC3 cells) reported that RGS2 negatively modulates the insulin secretion mediated by the glucose-dependent insulinotropic polypeptide (GIP) through the GIP receptor desensitization, which could suggest a potential role in conditions associated with diabetes in humans and animal models [15].

In this context, the role of RGS2 in insulin signaling in humans has been related to specific genetic variations. One example is the RGS2 C1114G polymorphism found in male humans, which is associated with a higher body mass index (BMI) [16]. Whether this situation results in increased insulin resistance is still unknown and suggests a case of sexual dimorphism. Interestingly, another study found that the C to G substitution at position −391 in the RGS2 promoter increases RGS2 expression in adipocytes and is associated with metabolic syndrome in white European men [17]. Together, this evidence implicates RGS2 as a candidate in developing metabolic syndrome and its associated factors in T2DM. However, to date, there is no experimental indication of a direct relationship between changes in the expression or activity of RGS2 with insulin resistance.

Although it is well established that the development of insulin resistance is a multifactorial process, in the present report, we have studied the role of RGS2 in humans, and we show that increasing the expression of RGS2 resulted in insulin resistance in human endothelial cells and a clear correlation between uncontrolled T2DM and RGS2 expression levels in human platelets.

## 2. Results

### 2.1. PA Increased RGS2 Expression in Human Umbilical Vein Endothelial-CS (HUVEC-CS) Cells

**Figure 1:**
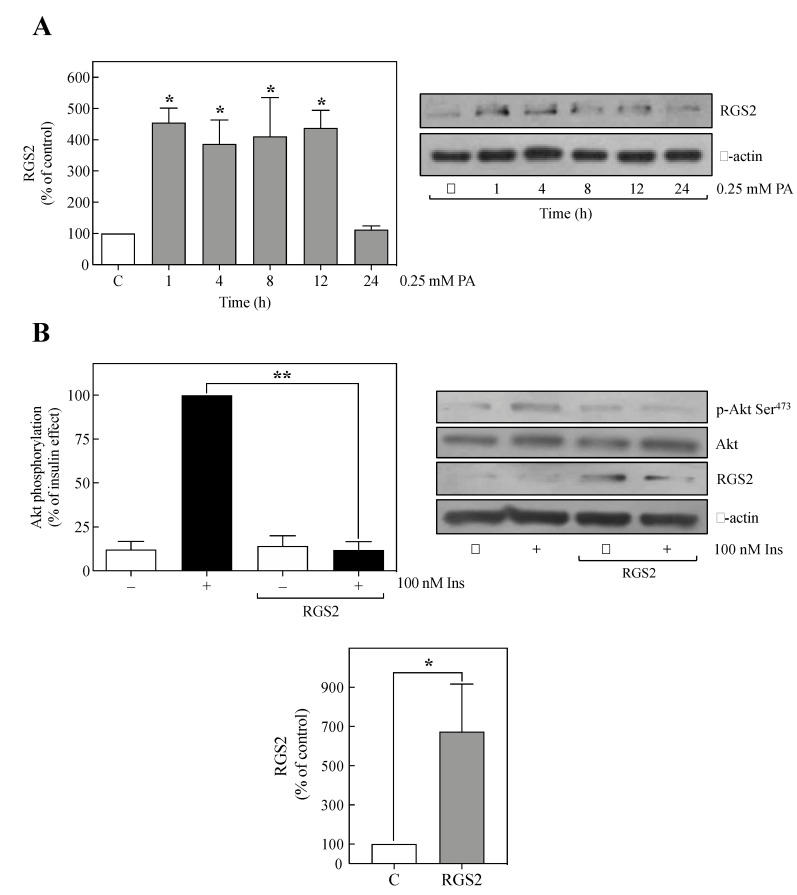
Increase in regulator of G-protein signaling 2 (RGS2) expression and insulin resistance. (**A**) Human umbilical vascular endothelial-CS (HUVEC-CS) cells were preincubated in the control medium (with dimethyl sulfoxide (DMSO)) for 24 h or medium containing 0.25 mM palmitic acid (PA) at different times before being harvested. Total cell lysates were separated by SDS-PAGE and analyzed by immunoblotting with anti-RGS2. Quantification of 3–6 independent experiments is expressed as mean ± S.E.M. The lower panel shows representative immunoblots. Western blots were also probed for total anti-β-actin as a loading control. (**B**) In the absence of PA, HUVEC-CS cells transiently transfected with human RGS2 were stimulated with 100 nM insulin for 10 min before being harvested. Total cell lysates were separated by SDS-PAGE and analyzed by immunoblotting with anti-p-Akt Ser^473^ (upper graph). RGS2 expression levels from the experiments described above were quantified and plotted on a bar graph as the mean ± S.E.M. (bottom graph). The right panel next to the upper graph shows representative immunoblots. The data is representative of four independent experiments. Western blots were also probed for total Akt or β-actin. (**A**,**B**) * *p* < 0.05 vs. C. (**B**) ** *p* < 0.05 vs. Ins (+). C, control; Ins, insulin; PA, palmitic acid.

Different reports have established the role of alteration in RGS2 expression in the genesis of metabolic disorders [14,18,19]. To determine whether insulin resistance state in HUVEC-CS cells leads to alterations in RGS2 expression, we performed a time course of RGS2 expression for cells incubated with 0.25 mM PA, since we have used previously this concentration [6], which is adequate to induce insulin resistance in HUVEC-CS cells (Figure S1). As shown in Figure 1A, PA increased RGS2 expression approximately 3.5-fold over control, at the earliest time tested (1 h), and remained elevated for up to 12 h and then abruptly declined to the basal level at 24 h. These results suggest that PA increased RGS2 expression in HUVEC-CS cells, similarly, as it occurs in the in vivo mouse model fed with a HFD [14].

### 2.2. The Overexpression of RGS2 Inhibited Insulin-Induced Akt Phosphorylation

Since PA increased RGS2 expression, we decided to determine whether RGS2 overexpression without PA induces insulin resistance. To this end, the inhibition of insulin-induced Akt Ser^473^ phosphorylation was used as a molecular indicator of insulin resistance in RGS2 overexpression. Thus, human *rgs2* transiently transfected in wild type HUVEC-CS cells resulted in a significant 6-fold increase in RGS2 protein expression (Figure 1B, bottom graph). Interestingly, the insulin-induced Akt phosphorylation was inhibited by RGS2 overexpression without any effect on the basal phosphorylation level or the amount of Akt (Figure 1B, upper graph). Therefore, these results suggest that an increase in the RGS2 expression without any addition of PA interferes with insulin ability to trigger Akt phosphorylation.

### 2.3. Platelets from T2DM Patients Displayed Increased RGS2 Protein Expression

**Figure 2:**
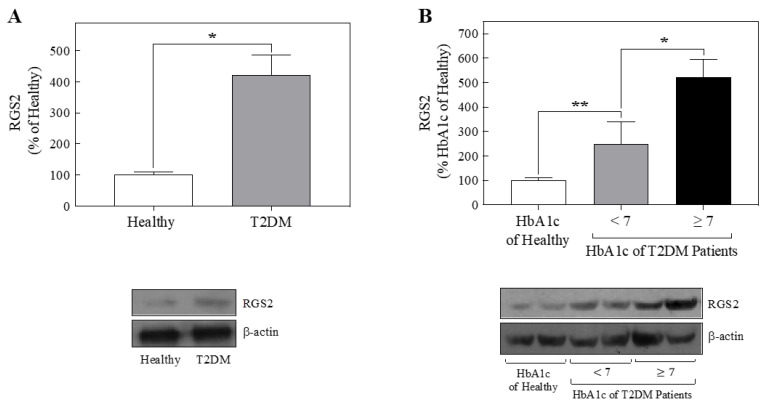
T2DM patients have increased levels of RGS2 protein. (**A**) Cell lysates from platelet-enriched fractions from blood samples of healthy individuals (*n* = 6) and T2DM patients (*n* = 11) were separated by sodium dodecyl sulfate-polyacrylamide gel electrophoresis (SDS-PAGE) and analyzed by immunoblotting with anti-RGS2. (**B**) Cell lysates from platelets-enriched fractions from blood samples of healthy individuals (glycated HbA1c < 5.7% *n* = 6) and T2DM patients with increasing percentages of glycated HbA1c (<7% *n* = 4 and ≥7% *n* = 7) were separated by SDS-PAGE and analyzed by immunoblotting with anti-RGS2. The graph indicates the mean ± S.E.M. Lower panels for (**A**) and (**B**) are representative immunoblots. Western blots were also probed for β-actin as a loading control. (**A**) * *p* < 0.05 vs. Healthy individuals, (**B**) * *p* < 0.05 vs. T2DM patients with glycated HbA1c < 7%; ** *p* < 0.01 vs. HbA1c of Healthy (control group).

Since the overexpression of RGS2 inhibited insulin signaling in human endothelial cells, the next step was to quantify RGS2 expression levels in platelets from T2DM patients and compare them with those obtained from healthy people. Remarkably, a threefold increase in RGS2 expression was observed in T2DM patients compared with those obtained from healthy individuals (Figure 2A).

### 2.4. Principal Component Analysis Displayed a Positive Correlation between the RGS2 Protein Expression Level and the Percentage of Glycated Hemoglobin

The positive correlation between the percentage of glycated hemoglobin (HbA1c) level and a higher risk of developing complications associated with diabetes was established in the Diabetes Control and Complication Trials for T2DM patients [20,21,22,23]. These findings have positioned HbA1c as an indicator of long-term glycemic control and generate a therapeutic guide for all diabetic patients. T2DM patients are within therapeutic goals when they have less than 7% HbA1c. As an initial analysis to study the correlation between RGS2 expression levels in platelets and HbA1c amount, HbA1c 7% was the threshold to separate T2DM patients two groups (Table 1) [20,21].

The results showed that RGS2 expression levels were grossly correlated with the percentage of glycated hemoglobin; patients with HbA1c < 7% had RGS2 expression approximately 1.5-fold higher than healthy people, and patients with HbA1c ≥ 7% showed RGS2 expression approximately 4-fold larger than healthy people (Figure 2B).

**Figure 3:**
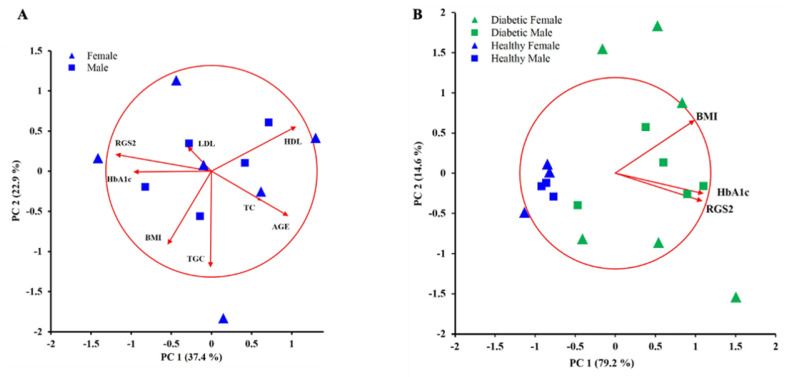
Correlation biplots are showing the variability explained by the new sets of variables conforming PC1 and PC2. (**A**) Exploratory principal component analysis (PCA) of nine different variables for eleven T2DM patients; scatter plot showing the position for each one of the T2DM patients within PC1 and PC2 dimension. PC1 was negatively formed by RGS2 and HbA1c, and positively by HDL and age, while TGC and BMI formed PC2. There is no apparent clustering of T2DM subjects, except that seven T2DM subjects were distributed along PC1 (between 0.5 and −0.5 of PC2). Blue triangles correspond to T2DM females; blue squares correspond to T2DM males. (**B**) PCA analysis for the same diabetic subjects shown in (**A**) and RGS2, HbA1c, and BMI for six healthy donors (in blue). In this case, all these three variables correlated heavily with PC1. The latter clustered at the low end of RGS2 and HbA1c, while T2DM subjects clustered at the high end of RGS2 and HbA1c. In this sample, it was found that T2DM females displayed a large variability in BMI compared with diabetic males (see Table 1). Green triangles correspond to T2DM females; blue triangles correspond to healthy females. Green squares correspond to T2DM males; blue squares correspond to healthy males.

An unbiased approach was to use exploratory principal component analysis (PCA) to find correlation patterns among nine observed variables derived from eleven T2DM subjects. The correlation biplot shows that the first two components out of eight explained the 60.3% variability in this data set (Figure 3A). The main components in PC1 were RGS2 (−0.908), high-density lipoprotein cholesterol (HDL) (0.802), HbA1c (−0.737) and, age (0.726). The biplot figure also shows that RGS2 and HbA1c have a direct correlation, while HDL and age displayed an inverse correlation with RGS2 and Hb1Ac in T2DM patients regardless of their gender. Moreover, the RGS2 level was predicted by a linear combination of these parameters (HbA1c, HDL, and age) with an adjusted R^2^ = 0.891. This correlation was the case for 7 out of the 11 patients (P3, P8, P9, P10, P11, P13, P17, see Table 1). P6 did not cluster with the mentioned seven patients because he presented both a low BMI (21.73) and HbA1c (5.7). The remaining three patients that did not distribute within PC1 were those located at the extreme values for triglycerides (TGC) levels, which was the primary variable making the second factor (−0.910). P4 had the lowest TGC level, while P2 and P14 presented the highest TGC levels (see Table 1).

Since the analysis shown in Figure 3A displayed a clear correlation between the RGS2 expression level and HbA1c, we decided to include the RGS2 level for six healthy donors. In this case, the correlation biplot explained 93.9% of the variability. The three variables defining PC1 with correlation coefficients above 0.7 were HbA1c (0.925), RGS2 (0.912), and BMI (0.831). Healthy donors clustered together at the low end of PC1, while T2DM subjects clustered at the high end of PC1, except for P6 and P9 that had very low BMI, and for this reason, they were close to healthy subjects. Interestingly, BMI displayed a more considerable variability in diabetic patients than in healthy donors. The biplot shows that T2DM females presented a large variability in the BMI variable. In this analysis, the age variable showed a lower correlation than BMI when including healthy donors; and since age displayed an inverse correlation with RGS2 expression (Figure 3A), for this reason, age was not considered for this new analysis. Figure 3B shows that the RGS2 level and HbA1c are still correlated even when healthy people are included with T2DM subjects.

## 3. Discussion

The correlation between the increase in RGS2 protein expression, reduction in insulin signaling, and T2DM was investigated in the present study. The results show that PA-induced a marked increase in RGS2 expression. Interestingly, in the absence of fatty acids, the overexpression of RGS2 in HUVEC-CS cells was enough to inhibit the insulin-induced Akt phosphorylation, in the same way that PA does [6]. Thus, the results suggest that an elevation in the expression of RGS2 protein level is sufficient to inhibit insulin signaling in human endothelial cells.

The RGS family members have been shown to play essential roles in cardiovascular physiology, energetic metabolism, and the regulation of inflammatory processes. RGS2 belongs to the largest family of RGS proteins, the B/R4 family, in which RGS4, RGS5, and RGS16 are also included. These four regulators play critical roles in the CNS [24,25], and recent evidence also indicates that they are implicated in regulating diverse metabolic processes, including energy homeostasis and insulin secretion [26,27,28]. Particularly RGS4, RGS5, and RGS16 deregulation or decrease in its expression in knockout or knockdown models generates severe metabolic disorders that include increased concentrations of circulating FFA, hepatic steatosis, decreased insulin secretion by pancreatic β-cells, and glucose intolerance, conditions associated with insulin resistance, and T2DM [26,27,28].

In contrast, RGS2 has shown a different behavior pattern in regulating metabolic functions [14]. As we show here, there is a clear correlation between insulin resistance and increased levels of RGS2 under the conditions of a high-fat diet. Likewise, in RGS2 knockout mice, it has been observed an increased insulin sensitivity. Therefore, although it has been shown the role of these RGS proteins in the regulation of metabolism, their effects are opposite: while the increase in RGS4 and RGS16 expression would substantially improve the conditions of insulin resistance and T2DM [26,27], the increase in RGS2 determine a state of insulin resistance and also, as observed in our study, its increase in T2DM patients constitutes a novel finding that could be considered as a hallmark of this condition.

Previous studies have shown that an HFD induces insulin resistance and metabolic alterations in animal models; also, in this context, it has been demonstrated that mice fed a HFD presented an increased RGS2 expression [14]. Nevertheless, this effect had not been studied in human tissue models. To elucidate whether saturated fatty acids induce an elevation of RGS2 expression in human cells, a time course with 0.25 mM PA in HUVEC-CS cells was studied. Our data revealed that PA alone promoted a dramatic increase in RGS2 protein expression. Although previous studies have shown an association between elevated fatty acids and increased RGS2 protein levels in mice fed with a HFD [14,29,30], in the present study, it was found that PA alone is sufficient to increase RGS2 expression.

Although the elevated RGS2 biological implication is still unclear, our data show that human RGS2 overexpression in HUVEC-CS cells is sufficient to cause a significant decrease in insulin-induced Akt Ser^473^ phosphorylation, and the presence of high concentrations of saturated fatty acids was not required to induce this insulin resistance state. Therefore, it can be concluded that increased RGS2 protein expression might impair insulin signaling in HUVEC-CS cells. Furthermore, in agreement with our results and with the idea that RGS2 induces insulin resistance, it has been reported that those mutations resulting in increased RGS2 expression levels have been associated with metabolic syndrome and insulin resistance [17]. Interestingly, it has been demonstrated that *rgs2^−/−^* mice present increased insulin sensitivity and metabolic activity derived more from a higher energy consumption from metabolism than physical activity, suggesting that RGS2 may play an essential role in regulating the basal metabolism [14].

Regarding the mechanism associated with the increase in the RGS2 expression level by PA in HUVEC-CS cells, there is the possibility that the GPR40 receptor activation mediates this effect since its presence has been identified in vascular cells, including HUVEC cells. GPR40, also called free fatty acid receptor 1 (FFAR1), is a G protein-coupled receptor (GPCR) activated by long-chain fatty acids, such as PA [31]. Activation of GPR40 receptor with cognate ligands elevates intracellular Ca^2+^ levels and increase PKC activity via coupling to Gα_q/11_ [31,32,33], a pathway associated with RGS2 up-regulation [34,35,36]. Consistently with the idea that GPR40 may mediate the observed effects of PA on RGS2 expression, Hernandez-Caceres, et al. [32] recently demonstrated that PA reduces insulin sensitivity through GPR40 activation in hypothalamic neuronal cells. In this regard, GPR40 seems to be a good candidate to mediate the PA-mediated RGS2 up-regulation.

However, it is important to consider that long-term FFA exposure, as the PA in the present work, could enter the cells triggering oxidative stress and endoplasmic reticulum (ER) stress, two major contributors to insulin resistance [6,37]; the induction of oxidative stress also could be a critical factor for the increased PKC activity [38].

Concerning how a PA-induced increase in RGS2 protein level in HUVEC-CS cells promotes an insulin resistance state, it has been proposed that RGS2 negatively regulates the previously demonstrated Gα_q/11_-mediated insulin signaling and that the overexpression of WT-RGS2 inhibits the insulin-stimulated GLUT4 translocation in 3T3-L1 adipocytes [39]. Moreover, it has been shown that thiazolidinediones, which decrease insulin resistance, reduce RGS2 expression [40]. Furthermore, RGS2 has been described as a critical component of the cellular stress response, including the heat shock response, oxidative stress response, and the endoplasmic reticulum (ER) stress response pathways [41,42]. In this way, RGS2 can also be a component of the PA-induced ER stress response that we previously found associated with insulin resistance in HUVEC-CS cells [6].

In the present work, we demonstrated that T2DM patients with HbA1c above the threshold of 7% displayed higher RGS2 expression in platelets. Remarkably, this characteristic did not correlate with the presence of dyslipidemia, particularly TGC levels. Our specific aim was to identify patterns of correlation among nine different variables that have been associated with T2DM subjects. Diabetic patients did not show any particular clustering pattern. Surprisingly, BMI displayed a poor correlation with HbA1c and correlated better with TGC levels, and these two variables ran orthogonally to RGS2 and HbA1c. The only variable that showed a clear positive correlation with HbA1c was RGS2, independently of age and sex in T2DM subjects.

Although it is not new that high HDL levels have an inverse correlation with diabetes, we did not expect that age was inversely correlating with the RGS2 level in T2DM patients based on the RGS2 knockout mice model. The reason for this inverse correlation between RGS2 and age is that the higher RGS2 expression levels were found in young T2DM patients and not in the older ones. It appears then that young T2DM with high RGS2 levels have poorly controlled diabetes. When RGS2 levels from healthy donors were included, a clear correlation was obtained between RGS2 and HbA1c, with clustering of healthy donors in low levels of RGS2 and low levels of HbA1c with low levels of BMI, while T2DM patients showed high levels of both RGS2 and HbA1c. In this case, the BMI variable was the source of variability among the T2DM subjects.

Interestingly, the T2DM patients located at both ends of the BMI variable were females. The limited number of T2DM patients involved in the present study does not allow to be conclusive about the females being T2DM with low BMI but is a trait that will be important to explore in future studies to warn the general population that obesity is not the only variable to consider for the development of T2DM. Nevertheless, this T2DM exploratory PCA study, together with the observation that RGS2 overexpression decreases insulin signaling in HUVEC-CS cells, supports the need for studying how RGS2 relates to diabetes in humans. It is essential to highlight that correlation does not imply causation; nevertheless, overexpression of RGS2 leads to reduced insulin signaling as shown in this work, and the RGS2 deficient mice are resistant to age-related weight gain [14], then it becomes imperative to explore whether a high level of RGS2 leads to a condition of poorly controlled glycemia in young T2DM subjects.

These data collectively lead us to propose that the increase in RGS2 expression is enough to trigger insulin resistance in endothelial cells, and uncontrolled glycemia in T2DM patients correlates with a strong increase in RGS2 protein expression level in platelets. In conclusion, this study provides further work on the role played by RGS2 in T2DM patients.

## 4. Materials and Methods

### 4.1. Materials and Antibodies

Dulbecco’s modified Eagle’s medium (DMEM), palmitic acid (PA) (≥99% pure), and fatty acid-free bovine serum albumin (FAF-BSA) were from Sigma Aldrich. Fetal bovine serum (FBS) was from ByProductos. Attachment factor protein (AF) 1X and Lipofectamine 2000 were from Life Technologies. Anti-phospho-Akt Ser^473^ (anti-p-Akt Ser^473^) (sc-7985), anti-Akt (sc-8312), anti-RGS2 (sc-9103) and anti-β-actin (sc-1616) were from Santa Cruz Biotechnology. The full-length human RGS2 3xHA-tagged cDNA clone (ID RGS020TN00) in the pcDNA3.1+ vector was obtained from the cDNA Resource Center (www.cdna.org).

### 4.2. Cell Culture and Transfection

The human umbilical vein endothelial cells (HUVEC-CS) were obtained from ATCC. HUVEC-CS cells were grown at 37 °C in a humidified environment of 95% air, 5% CO_2_, in DMEM supplemented with 20% FBS, 100 μg/mL streptomycin, and 100 units/mL penicillin in plastic P-100 Petri dishes pretreated with AF for 30 min [6]. All experiments were carried out using HUVEC-CS cells between passages 2 and 13 [6]. For the experiments, HUVEC-CS cells were subcultured in either 6- or 12-well plates (previously pretreated with AF for 30 min) until the cells reached 80% confluence and then cultured with serum-free DMEM for another 6 h before PA or insulin treatments [6]. For RGS2 overexpression experiments, HUVEC-CS cells were seeded at 2.5 × 10^4^ cells/well in 6-well plates previously treated with AF for 30 min and cultured for 3 days. Then cells were transfected with RGS2 3xHA tagged cDNA (1 μg/well) using Lipofectamine 2000, as previously described [6].

### 4.3. Study Population

#### 4.3.1. Ethical Statement

The protocols carried out in the present study were previously approved by the Hospital General 5 de Diciembre of ISSSTE Mexicali, Mexico (Circular letter number 0985/2017), following the principles of the Declaration of Helsinki, as revised in 2000. All subjects, T2DM patients and healthy people, were recruited after a check-up with their Family Physician, obtaining informed written consent to carry out a pilot study.

#### 4.3.2. Subjects

In the present double-blind study, patients with diabetes mellitus were considered. Later from this group, 11 subjects diagnosed with T2DM according to the American Diabetes Association standards of medical care in diabetes were selected [43], including patients with fasting plasma glucose (FPG) ≥ 126 mg/dL (7.0 mmol/L), HbA1C ≥ 6.5%, or patients with random plasma glucose ≥ 200 mg/dL (11.1 mmol/L), with classic hyperglycemia symptoms (Table 1). The control group included 6 healthy individuals with FPG < 100 mg/dL, HbA1C < 5.7%, BMI < 25%, no comorbidities, neither pathologic signs nor symptoms of both genders and ages between 19–60. Congestive heart failure, acute or chronic renal failure, type 1 diabetes mellitus, acute or chronic pancreatitis, acute or chronic liver disease, and pregnancy were considered exclusion criteria.

#### 4.3.3. Measurement of Serum Parameters

Plasma levels of total cholesterol (TC), TGC, HDL, and low-density lipoprotein cholesterol (LDL) were measured from 5 mL of whole venous blood samples using a Roche/HITACHI Cobas 6000 analyzer (Roche Diagnostics, Basel, Switzerland). Blood serum levels of HbA1c were determined from 5 μL of fasting blood samples using the COBAS b 101 analyzer (Roche Diagnostics, Basel, Switzerland).

### 4.4. Preparation of Human Plasma and Platelets Purification

Ten milliliters of peripheral blood were transferred into sterile polypropylene tubes containing sodium citrate (Vacutainer System, BD Biosciences). Fractions enriched in platelets were obtained as described previously [44]. Plasma was carefully aspirated from the pellet and centrifuged at 800× *g* for 15 min at 4 °C to obtain plasma fractions enriched in platelets. Finally, the fractions enriched in platelets were lysed, and the protein levels of each sample were determined by the micro-Bradford protein assay [45].

### 4.5. Immunoblot Analysis

After treatments, media was removed by aspiration, and the cells were washed once with ice-cold PBS and lysed with 100 μL of Laemmli sample buffer, and the cell lysates were used for immunoblot assays, as previously described [6]. Thus, cell lysates were thawed to room temperature and briefly sonicated, heated at 99 °C for 5 min, and centrifuged at 14,000 rpm for 5 min. The supernatant was electrophoresed on SDS-PAGE (8 or 10%) gels and transferred to PVDF nylon membranes. Blots were incubated overnight at 4 °C with primary antibodies and washed three times with TBST before probing with horseradish peroxidase-conjugated secondary antibodies for 1 h at room temperature. The blots were then visualized with Millipore Immobilon Western HRP substrate peroxide solution. When necessary, the membranes were stripped for reuse with Restore Western blot stripping buffer (Thermo Fisher Scientific, Waltham, MA, USA) for 30 min, and then the complete removal of the primary antibodies was ensured. Quantification of immunoblot films was carried out with ImageJ software version 1.53h (National Institutes of Health, Bethesda, MD, USA). Blots were reprobed with antibodies against the corresponding total protein to assure equal loading of proteins. In the case of RGS2, the loading control was β-actin.

### 4.6. Statistical Analysis

Average intensity from Western blot films was analyzed using one-way ANOVA with Dunnett’s post-test using the GraphPad PRISM^TM^ version 8.4.3 for macOS (GraphPad Software, Inc., San Diego, CA, USA). In all cases, a *p*-value < 0.05 was considered statistically significant. This data was normalized using either the control or the insulin responses, and the mean ± S.E.M. was plotted for at least three separate experiments [6]. Figures show representative blots.

### 4.7. Multidimensional Correlation Analysis

Multidimensional correlation analysis was carried out using PCA implemented in the plugin Analyse-it™ for Microsoft’s Excel™ 5.30.5. Nine different variables from eleven T2DM subjects and only four variables from healthy donors were analyzed with exploratory PCA [46]. These parameters were sex, BMI, RGS2 levels determined by western blot, HbA1c, age (years), TGC, TC, HDL, and LDL (see Table 1). Variables with correlation coefficients higher than 0.7 were considered relevant for determining both PC1 and PC2. The scree plot is not shown, but the percentage of total variance explained by the first two factors is indicated in parenthesis in the biplot figures. No rotation was applied to the analysis, and the normalization used was defined by variance. The correlation among the different variables was displayed using a correlation biplot.

## 5. Conclusions

Palmitic acid, a saturated fatty acid, inhibits insulin signaling in vascular endothelial cells, and this action is associated with an increased expression of RGS2 protein, one of many regulators of G protein signaling. Moreover, the overexpression of this protein alone was enough to inhibit insulin signaling. These data suggest that RGS2 protein associates with decreased insulin signaling at the cellular level. Remarkably, we have found that the amount of RGS2 protein from platelets correlates with uncontrolled hyperglycemia in type II diabetic patients, particularly those at a young age. Hence, increased levels of RGS2 is a feature associated with poorly controlled type 2 diabetes mellitus. Understanding how RGS2 is associated with diabetes could become this protein into a new therapeutic target for those patients with hard to control glycemia.

## Figures and Tables

**Table 1:** Body Characteristics and Serum Blood Levels for Different Tests in T2DM Subjects.

Patients with Type 2 Diabetes Mellitus										Healthy					
ID	Gender	Age	HbA1c	RGS2 Densitometry	BMI (Kg/m^2^)	TC (mg/dL)	TGC (mg/dL)	HDL (mg/dL)	LDL (mg/dL)	ID	Gender	Age	HbA1c	RGS2 Densitometry	BMI (Kg/m^2^)
P3	Female	60	5.6%	138.4991	31.63	193	202	47	105.6	1	Male	25	5.0	100	20.4
P6	Male	59	5.7%	237.5115	21.73	161	127	52	83.6	2	Male	27	4.8	103.2773	19.9
P9	Female	58	6.1%	270.1421	20.31	260	205	73	70	3	Female	23	4.8	100	21.5
P14	Female	58	6.2%	356.1189	37.28	199	565	36	50	4	Male	23	5.4	116.2006	20.2
P13	Male	67	7.3%	312.7119	30.82	172	108	55	95.4	5	Female	24	5.0	100	21.2
P4	Female	29	7.4%	568.4774	26.17	163	74	54	94.2	6	Female	24	4.4	93.20259	17.3
P8	Female	52	7.5%	465.9215	35.086	148	120	56	68						
P10	Male	50	7.6%	440.2003	30.47	147	90	42	87						
P11	Male	48	7.9%	681.8213	32.62	151	210	28.9	80.1						
P17	Female	35	9.5%	856.7031	29.31	173	232	25	101.6						
P2	Male	57	10%	330.433	30.035	247	355	43	133						

Note: HbA1c, glycated hemoglobin; BMI, body mass index; TC, total cholesterol; TGC, triglycerides; HDL, high-density lipoprotein cholesterol; LDL, low-density lipoprotein cholesterol.

## Data Availability

The data presented in this study are available on request from the corresponding author. The data are not publicly available due to privacy and ethical restrictions.

## Supplementary Materials

The following are available online at https://www.mdpi.com/2218-1989/11/2/91/s1. Figure S1: Effect of PA on the insulin signaling pathway and RGS2 expression.
