# Supplementary material for: The Increased Expression of Regulator of G-Protein Signaling 2 (RGS2) Inhibits Insulin-Induced Akt Phosphorylation and Is Associated with Uncontrolled Glycemia in Patients with Type 2 Diabetes
Source: Metabolites. 2021 Feb 5;11(2):91. doi: 10.3390/metabo11020091 (PMC7915073; doi:10.3390/metabo11020091)
Supplement: Supplementary file 1 [file metabolites-11-00091-s001.pdf]

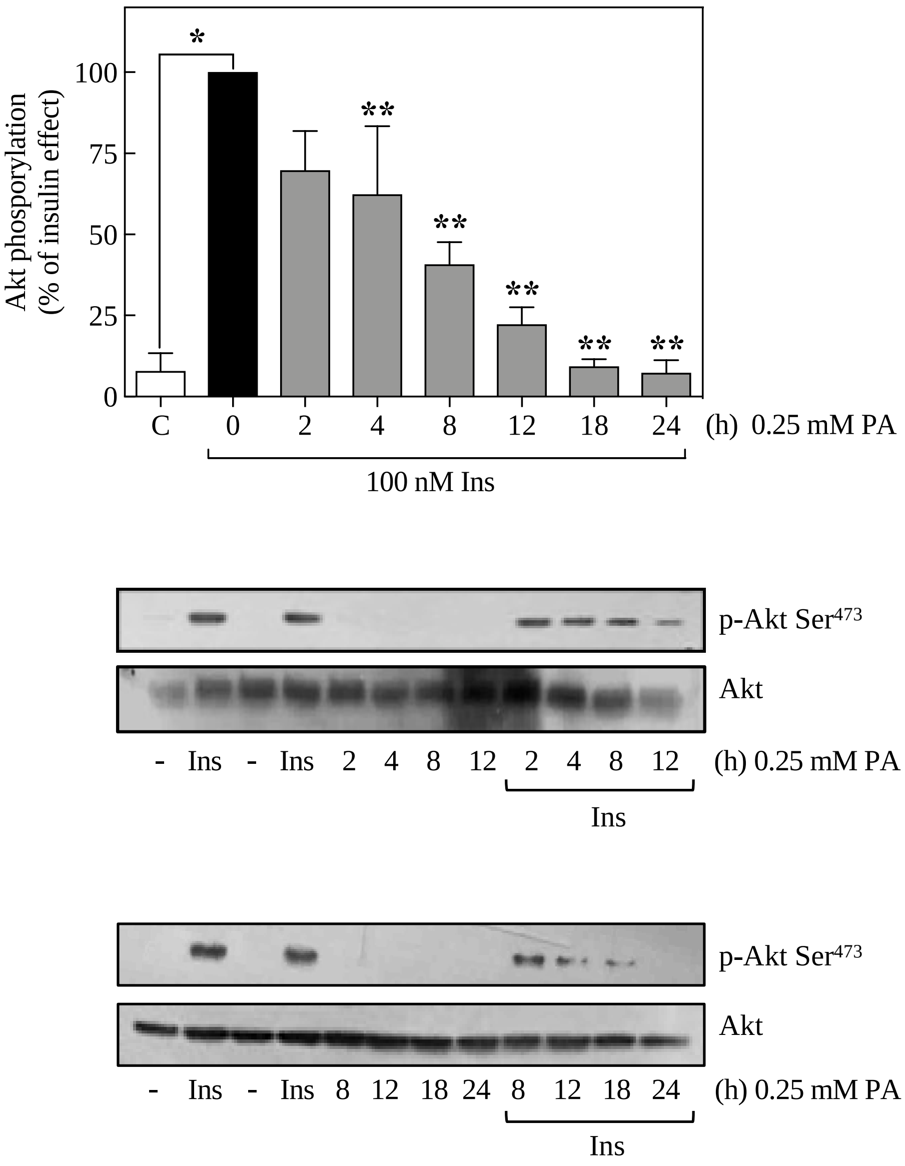

Figure S1. Effect of PA on the insulin signaling pathway and RGS2 expression. HUVEC-CS cells were preincubated in a control medium (with DMSO) for 24 h or medium containing 0.25 mM PA at 37 °C for the indicated times and stimulated with 100 nM insulin for 10 min before being harvested. Total cell lysates were separated by SDS-PAGE and analyzed by immunoblotting with anti-p-Akt (Ser^{473}). Lower panels show representative immunoblots. The graph is representative of three-six independent experiments and is expressed as mean ± SEM. Western blots were also probed for total Akt showing equal loading. *$p < 0.05$ *vs* C; **$p < 0.05$ *vs* Ins (0). C, control; Ins, insulin; PA, palmitic acid.
